# Beyond chatbots: generative AI as public health infrastructure and a new Digital Social Determinant of Health

**DOI:** 10.3389/fpubh.2026.1910815

**Published:** 2026-07-31

**Authors:** P. G. Suraj, S. Clemence Jenifer, R. Joice Swarnalatha, R. Sangeetha

**Affiliations:** Vellore Institute of Technology, Vellore, India

**Keywords:** AI governance, Digital Public Health, generative artificial intelligence, public health infrastructure, Social Determinants of Health

## 1 Introduction

In the recent past, generative artificial intelligence (AI) has gone from being a novelty to a powerful force that affects people's ways of seeking information, decision making, and engaging with institutions. In healthcare and public health, the focus has been on leveraging AI chatbots for delivering health information, patient support, and conversational assistance (1, 2). These have shown great potential in making things more accessible, engaging and facilitating digital health applications. The chatbot story, however, may all but mask a more fundamental shift in the way things are going. Generative AI is becoming more of a go-between for people and health information. Today, millions of people are consulting AI systems to interpret symptoms, assess health risks, gain insights into health conditions, access mental health resources, and understand healthcare options. People are using AI systems to help them interpret symptoms, evaluate health risks, understand health conditions, seek mental health resources, and understand healthcare options. This momentum is expected to continue, and generative AI is transforming from a digital instrument into a societal infrastructure that is impacting the health knowledge ecosystem as it is developed, delivered, and used. This shift is part of an overall trend in public health to acknowledge the influence of digital technologies on population health. The Social Determinants of Health (SDOH) have long been recognized as socioeconomic and environmental factors that influence health outcomes, such as education, income, housing, work, and social supports (3). Recently, researchers and international public health bodies have suggested that the opportunity to use digital access, connectivity, digital literacy, and use digital health services are new factors shaping inequities in health. However, current discussions have mainly addressed the infrastructure and access to technology, but not the increasing role of smart systems that actively interpret, synthesize and personalize health information. This ever-changing environment presents a significant conceptual disconnect around the place of Generative AI in modern public health. In this opinion article, the authors argue that boiling generative AI down solely to an “app store of chatbots” leaves the transformative potential of generative AI understated when it comes to public health. Instead, generative AI should be seen as an emerging public health infrastructure that has the capacity to impact at scale on population health outcomes. Also, reliable health data and information provided by AI can become a new Digital Social Determinant of Health (DSDH). Given that education, income and housing impact health outcomes, access to a reliable AI system could have an impact on health literacy, health behaviors, healthcare navigation, health preparedness and health equity. In this article, we are not suggesting that having reliable access to Generative AI is a new determinant of health that sits outside of the public health domain, but rather that it is the next step in the evolution of digital determinants of health. This view extends SDOH frameworks with intelligent, AI-enabled access to health knowledge, introducing a conceptual basis for analysis of how Generative AI can transform health equity, public health communication and population health resilience in the increasingly digital societies we live in. This is especially true of Digital Public Health, which goes beyond the scope of clinical settings to include prevention, population health promotion, community resilience, and health resource equity. This article aims to present the opportunities of generative AI to public health, its long-term impact on health equity, public health preparedness, and population wellbeing as both public health infrastructure and a potential Digital Social Determinant of Health.

## 2 The emergence of AI-mediated health societies

Traditionally, the dissemination of public health information has been done via health care providers, government agencies, schools, and mainstream media. The digital revolution turned this all upside down and made it possible to access health information online directly. Generative AI is a paradigm shift in the health information landscape, and could one of the most impactful digital health transformations of our time. Recently, there has been a substantial growth of AI-driven conversational systems in healthcare and various other industries (4). Modern-day healthcare Chatbots can now help users interpret their symptoms, get information about medicines, preventive care advice, health education, and mental health support (5). The use of these technologies has increased over time and become part of health-seeking practices. This shift has more than a technological purpose. AI systems are becoming the first point of contact for many for health related questions. This change has changed the way health information is retrieved and understood. Instead of simply being told about from institutional sources, people now speak to AI systems that can respond uniquely to them (6). This new AI-enabled health landscape opens up new opportunities for public health involvement. AI systems can be used to deliver information in multiple languages, adapt to different levels of literacy, and offer round-the-clock information, irrespective of geographical location. These can augment the effectiveness of public health interventions dramatically. However, there are significant questions that need to be addressed with the increasing use of AI-driven interactions. Who will be in charge of the information produced by these systems? How do you know that it is accurate? What measures should be taken to stop the spread of misinformation? The main question now is: what will happen when access to reliable AI is not evenly distributed amongst populations? The quoted questions indicate that generative AI is not just a communication tool. Instead, it is now turning into a fundamental part of the modern health information landscape. AI-enabled health societies also have implications for the global health infodemic. In the event of a public health emergency, Generative AI can quickly create and share evidence-based information, thereby enhancing public health communication, and also help with health promotion. Meanwhile, these systems can also produce misleading, biased or false health information that can be widely disseminated. Thus, generative AI can be a force both for good and evil in health infodemics, underscoring the necessity to treat it as a public health infrastructure that needs to be governed and continually assessed.

## 3 Generative AI as a new Digital Social Determinant of Health

Traditional social Determinants of Health have been defined as income, education, employment, housing, and social support. All of these factors have an impact on individuals' health as they can access health care services and achieve good health (7). Social Determinants of Health are defined by the World Health Organization (8) as the conditions in which people are born, grow, live, work and age, noting that these conditions are influenced by the “distribution of power, resources and opportunities in society. Likewise, the Center for Disease Control and Prevention (CDC) identifies the following as essential factors that influence health outcomes: economic stability, education, healthcare access, neighborhood environment, and social context. These frameworks show that health is not only shaped by the health care system but also by factors that are beyond the health care system, such as structural and societal factors that affect the opportunities for health. These well-defined viewpoints have been reinforced in recent public health publications, and it is now acknowledged that digital determinants of health (DDOH) are important risk factors for access to health information, health service use, and health inequities, including access to digital technologies, internet connectivity, digital literacy, and digital health services (9). The Digital Social Determinant of Health (DSDH) is not intended to supplant current Social Determinant of Health (SDH) theory, but rather to be applied to the existing concepts. Education, income and social context remain important determinants in access and use of digital technologies. Trustworthy Generative AI will in turn depend on these factors to facilitate the way people access, understand, and use health information. Therefore, DSDH is not a separate pathway from SDOH, but a further one that helps to accentuate social inequalities and further harms health in digitally connected societies. As healthcare went digital, social determinants have been extended to include technological determinants that influence people's ability to interact with the health systems of the 21st century. Access to digital has become a key factor in health equity in recent years. In this opinion article, the authors suggest that generative AI could be the next step in this idea. In particular, having access to reliable AI systems could become a Digital Social Determinant of Health. Generative AI differs from previous digital determinants, which were mainly used for accessing health information online or accessing digital health services, by being able to interpret, synthesize, contextualize and personalize the health information through natural language interactions. Thus, while access to a reliable generative AI is not solely tied to internet connectivity or digital literacy, there are additional factors involved. It demonstrates a person's capacity to access understandable, contextually appropriate, evidence-based health information that fosters health literacy, health care navigation, and informed decision making. From this perspective, generative AI is an emergent aspect of current digital determinants and not a substitute for them. Users of sophisticated AI systems could gain from greater health literacy, more insight into preventive health practices, increased awareness of health risks, and more efficient interactions with the healthcare system. AI systems can help to streamline complex medical information and deliver tailored educational materials and support informed decision making (10). On the other hand, people without reliable access to AI technologies may be subject to new types of disadvantage. Access to AI-powered health resources might be hindered by limited digital literacy, insufficient technological infrastructure, and economic constraints. This means that entrenched inequalities in health may be exacerbated, if not reaffirmed. This view is in line with the general commentaries on digital inclusion in public health. Digital inequalities already impact on access to telemedicine, health information and digital interventions. If the benefits of generative AI are concentrated in technologically privileged populations, it could lead to an increase in inequalities. Also, the term ”AI divide" should be seen as a part of the digital divide. The AI divide can be defined as inequalities in access to trustworthy artificial intelligence (AI) systems, AI literacy, computational resources, language inclusivity and the ability to critically analyze AI-generated information, in addition to the traditional digital divide, which mainly refers to the differences in internet connectivity and access to digital technologies. Taking on the role of a Digital Social Determinant of Health does not mean that AI should be seen as a substitute for the other Social Determinants of Health. Instead, it recognizes that digital technologies now have a growing impact on people's ways of accessing health knowledge, understanding health risks and interacting with health systems. The accessibility, digital literacy, equitable deployment, interoperability, and governance of AI are thus key aspects to be built into future health equity strategies that must be addressed by public health policies (11). Generative AI serves as a bridge between human and health information. Generative AI is distinct from previous digital tools, which mainly provided access to information, by actively interpreting, synthesizing, and personalizing information that will impact health understanding, decision making and healthcare navigation. Its function thus goes beyond access to digital content and contributes to the opportunities for health itself. Therefore, the trustworthy access to Generative AI is a second-order digital determinant, building on existing digital determinants and literacies. People need to have the right access to the internet and skills to interact with it, before they can benefit from AI powered health support. Generative AI therefore should be understood as a “family of Digital Social Determinants of Health (DsdH) that builds on and continues to expand existing public health frameworks” (12). Access to AI can thus be expected to increasingly become a Digital Social Determinant of Health through its impact on health literacy, health service engagement, and ability to make informed health choices.

## 4 From chatbots to population intelligence systems

The Discussions today often see generative AI as a series of conversational tools. Though chatbot applications are still significant, this view does not capture the greater public health potential of the technology. Beyond individual interactions, public health systems need capabilities. Effective disease surveillance, emergency preparedness, risk communication, community health education and community engagement rely on good management and dissemination of information at the population level. It is now crucial to differentiate between Generative AI and predictive or analytical artificial intelligence. Traditional machine learning and predictive AI are used mostly for the analysis of structured data to reveal patterns, assess risk, predict disease outbreaks, and epidemiological surveillance. These models use statistical learning methods to make predictions, and are commonly used for outbreak detection, disease prediction and health risk modeling. Conversely, Generative AI is not intended for predictive modeling, but to understand, synthesize, generate, and communicate in natural language. Therefore, the benefits of Generative AI at the individual level should not be seen as a replacement for predictive AI, but as a complement to it within the overall framework of the public health system (13). Predictive AI, for instance, can use surveillance data and predict the outbreak of a new infectious disease; while Generative AI can quickly compile and synthesize reports from various sources of surveillance information, interpret complex epidemiological findings, create multilingual public health advisories, create health education content for different audiences, and translate technical evidence into actionable policy briefs for decision makers. These additional features allow Generative AI to improve information created by traditional analytical systems to be more accessible, usable and shared. Generative AI has capabilities that are unique to them and could help with these functions at scale. In a recent study, Branda et al. (14) identified the increasing importance of AI systems in public health emergencies. But, emergency response is just one application field. In addition to the role of emergency response, Generative AI can help public health agencies by consolidating, analyzing, and providing context to a variety of sources of unstructured data, such as surveillance data, scientific publications, policy documents, social media conversations, community feedback, and field reports. These systems can quickly create evidence summaries, detect emerging themes, enable multilingual communication and promote the timely dissemination of consistent public health messages using natural language synthesis. These types of capabilities are especially useful during a fast-changing public health event, when decision makers have to assess massive quantities of diverse data within short timeframes. When combined with existing population-level surveillance, health databases, communication platforms, and public health workflows, generative AI needs to be thought of as a Population Intelligence System that can augment population-level surveillance, communication, preparedness, and evidence-informed decision-making (15). The proposed Population Intelligence System should thus be considered a socio-technical system, not a mere AI application. In this ecosystem, while predictive analytics and traditional machine learning produce epidemiological intelligence out of structured data, Generative AI enables better interpretation, knowledge synthesis, contextualization, multilingual communication, and evidence translation. The value of human expertise is always critical in validating outputs and applying professional judgement and in using it to make decisions for the public good in an ethical manner. Importantly, the term “Population Intelligence System” does not mean that Generative AI carries out an autonomous disease surveillance or epidemiological forecasting. Instead, it is a digital universe that is integrated with predictive analytics that provide insights for the population, and Generative AI that helps interpret, contextualize, communicate, and translate knowledge. This holistic approach to Generative AI serves as a smart front-end for analytical results, seamlessly interfacing with public health workers, public health policymakers, and the general public. A Population Intelligence System combines numerous data sources, detects trends, develops specific communication plans, and informs decisions based on data. Conceptually, this is crucial as the special promise of Generative AI is to bring out analytical results and deliver an actionable, contextually aware, and comprehensible knowledge for different stakeholders. It's not prediction, but knowledge synthesis, contextual interpretation, and communication at scale. These systems could help improve public health surveillance of outbreaks, public health communication about health threats, countering public health misinformation, and public health community-based response coordination. For example, after identifying an outbreak using traditional surveillance systems, Generative AI could be used to create community-level alerts in multiple languages; deliver succinct scientific summaries to health care workers on the frontlines; develop targeted education materials for at-risk populations; respond to commonly asked public health questions; and provide evidence-informed, succinct briefing documents for policy makers. These abilities significantly improve the responsiveness and scalability of public health communication, but do not supplant the analytical underpinnings of a surveillance system. This infrastructure approach is similar to implementation science approaches that focus on integration instead of standalone technologies (13). Isolated AI applications are not likely to drive public health transformation. However, meaningful impact will only be achieved if AI is integral to the wider public health system architecture (16). Future investments, therefore, should focus on the interoperability, scalability, and integration of AI into existing systems. At the same time, it's essential to have strong governance structures in place to establish the complementary roles of predictive analytics, Generative AI, and human skills. It is, therefore, essential that public health agencies consider Generative AI as a complementing technology that enhances the translation, communication, and decision-making of public health, while allowing for human oversight and validation of evidence throughout the public health decision-making process. Generative AI's real power goes beyond simply responding to queries; it's about enhancing the intelligence and resilience of communities as a whole.

## 5 The AI divide: a new public health inequality

For centuries, public health officials have been aware of the impact of inequitable access to resources. The unequal distribution of income, disparities in education and access to health care remain as indicators of inequity in health outcomes globally. The same situation could arise with generative AI. The digital divide has been a traditional definition of information and communication technology access. The public health face may soon be a new challenge that it must address, namely the AI divide: the disparities in access to effective, reliable, and trustworthy AI systems. These can impact the access to AI-driven health information and decision support, potentially creating a competitive advantage for certain groups. People with advanced AI technologies can get instant health updates, personalized education and improved decision making support. The following benefits may not be available to everyone. This concern is especially important in low resource countries where skills are limited. It's also applicable for older people, for people who are marginalized and for people who are less digital literate. Accessibility and user engagement have repeatedly been shown to be crucial for the successful uptake of digital health technologies in research about digital health (17). If not carefully planned and appropriately executed to foster inclusion, AI-powered public health campaigns can only worsen disparities. Public health institutions need to therefore make a strategic commitment to tackle the AI divide. The investments needed to build digital literacy, increase community engagement, develop infrastructure, and design inclusively will be key to delivering equitable access to AI-powered public health benefits. AI accessibility needs, therefore, to be included in future public health policies, alongside broadband availability, digital literacy, and digital inclusion, when creating a system of health. Trust should therefore not only be seen as an ethical value, but as part of a broader understanding of how AI is integrated into public health systems to enable good governance, maintain public engagement, and ensure system resilience over time.

## 6 Trust as public health infrastructure

Technology cannot solve population health. Trust is a key component in the foundation of public health interventions. Public trust in institutions and sources of information is essential for vaccination campaigns, health promotion campaigns, emergency response activities, and disease prevention. This is the same thing with generative AI. There are also clear issues of transparency, explainability, accountability and bias in AI systems that have recently been identified (18). Ethical concerns are also highlighted in reviews of AI implementation, such as those regarding the risks of misinformation, privacy risks, and governance issues (19, 20). This is especially significant as generative AI frequently generates content that seems credible and accurate despite its inaccuracy. Technology which jeopardizes trust and spreads false information cannot be a component of public health systems. So, trust is a key component of public health infrastructure. Just as with the physical infrastructure, AI infrastructure needs governance frameworks that can provide reliability and accountability. All public health AI applications will need to have human oversight as the critical foundation. AI cannot be a substitute for public health workers. Rigorous oversight systems, limited review processes, clear results, and ongoing surveillance should be expected in all public health contexts for the use of AI (21). If generative AI is to have the legitimacy required to assist with population level health care initiatives, it must be protected in such a manner. Thus, trust must be seen as a prerequisite for all AI-based public health systems and not just an ethical issue. In the absence of trust in the reliability, transparency and accountability of AI systems, the potential benefits for the population health that generative AI promises will likely not materialize.

## 7 Discussion and conclusion

Healthcare and public health have been a hotspot for generative AI's rapid growth, as evidenced by the vast array of studies and discussions that have emerged. With its swift growth, generative AI has sparked considerable interest in the healthcare and public health sectors, with numerous studies and discussions emerging. Much of the existing discussions have been on chatbot applications and conversational interfaces. These technologies are useful; however, they are just a small part of the potential generative AI can make to public health. This is an opinion article that in its approach goes beyond the current debate on digital health transformation and offers a conceptual approach. It is not just a conversational tool, it can also serve as an enabling layer to facilitate knowledge synthesis, translation of evidence, multiple language communication, and informed decision-making within population health systems. The article also helps to extend the current public health landscape into a broader conversation about health infrastructure, digital inclusion, and health equity by situating Generative AI in that context. This opinion article contains two related arguments. These two propositions are interrelated conceptually. This will help to see the system-level role of Generative AI, and understand the implications of this technology at the population level for health equity as a Digital Social Determinant of Health. These visions, when combined, offer a complete picture of the possible consequences of Generative AI for the organization of public health systems and health opportunity distribution in increasingly AI-enabled societies. Generative AI should be viewed as a new public health infrastructure that can contribute to surveillance, public health preparedness, communication, and engagement with the public and community (22). Importantly, this infrastructure-centric view does not suggest that it's an alternative to traditional epidemiological modeling, predictive analytics, or public health expertise. Rather, it extends these capabilities by providing additional interpretation, communication and action on the intelligence derived from existing analytical systems at the population level. Second, access to reliable AI-facilitated health information can become a Digital Social Determinant of Health that impacts health literacy, health behaviors, health care navigation, and health equity. The idea of ensuring that the use of GenAI tools is considered a dynamic Digital Social Determinant of Health (DSDH) also builds on existing theories of digital health equity. Equitable access to accurate AI systems, alongside AI literacy and suitable governance, could prove to be increasingly vital factors in shaping population health outcomes as societies become more dependent on AI-mediated information environments. This lens offers a theoretical basis for future empirical studies of the impact of AI accessibility on health disparities among various groups and health care systems.
